# The relationship between social media addiction and emotional appetite: a cross-sectional study among young adults in Turkey

**DOI:** 10.1017/S1368980024000466

**Published:** 2024-02-15

**Authors:** Sumeyra Sevim, Damla Gumus, Mevlude Kizil

**Affiliations:** 1 Ankara Medipol University, Faculty of Health Sciences, Department of Nutrition and Dietetics, Altindag, Ankara, Turkey; 2 Hacettepe University, Faculty of Health Sciences, Department of Nutrition and Dietetics, Sıhhiye, Ankara, Turkey

**Keywords:** Social media usage, Social media addiction, Emotional appetite, BMI, Young adults

## Abstract

**Objective:**

The present study focused on the relationship between addiction to social media (SM) and emotional appetite in young adults.

**Design:**

Cross-sectional online survey.

**Setting:**

The Bergen Social Media Addiction Scale (BSMAS) and Emotional Appetite Questionnaire (EMAQ) were used, and the duration and frequency of SM tools usage were analysed.

**Participants:**

Five hundred and twenty-four participants (144 men and 380 women) aged between 18 and 25 years.

**Results:**

The mean of SM usage duration of participants was 3·2 ± 2·2 h per d along with a mean of BSMAS score of 16·1 ± 5·9. Concerning emotional appetite, the mean scores for positive and negative aspects of EMAQ were 4·4 ± 1·9 and 3·1 ± 1·2, respectively. The predominant SM tools were YouTube (92·6 %) and Instagram (90·3 %). Notably, a significant association was observed between SM addiction and the frequency of access to YouTube, Instagram, and Twitter, with addiction levels increasing as access frequency rose (*P* < 0·01).

**Conclusion:**

This study demonstrated a possible relationship between SM addiction and emotional appetite among young adults. However, further research with more prominent participants and a lengthier follow-up duration is necessary to elucidate how SM tools affect eating behaviour.

The escalating global use of social media (SM), an Internet-based communication tool, is a prevalent trend. Reports indicate that SM users exceeded 3·6 billion in 2020 and are projected to reach 4·41 billion by 2025. SM serves various functions, including communication, social interaction and image sharing^(1,2)^. While SM facilitates vital aspects like social connectivity and information access, it also entails inherent risks such as addiction and cognitive impairment. Research has extensively explored SM addiction, characterised by excessive or problematic usage, from diverse perspectives including mental, biological and social dimensions^(3,4)^.

Young adults are the primary active users of SM. They experience continuous exposure to diverse content and images^(5)^, which raises concerns about their susceptibility to eating and body perception disorders induced by media influences, making them a potentially vulnerable demographic in this context^(6)^.

Although existing research has highlighted connections between SM and eating disorders, such as restrained eating and eating-related concerns, there is a gap in the literature regarding the exploration of the interplay between SM addiction and emotional appetite. To address this void, the present study was conducted to assess the associations among SM usage, SM addiction and emotional appetite among young individuals.

## Materials and methods

### Study design and participants

The study was conducted through an online survey targeting individuals aged 19–25 years residing in Turkey. The online questionnaire was designed via Google.form, accessible via smartphones or computers. Exclusion criteria encompassed drug use, psychiatric disorders, undergoing eating behaviour therapy, pregnancy and lactation. Over a span of 2 months, a total of 588 participants were engaged. After addressing issues such as missing data (e.g. weight and height), incomplete responses to scales, and inconsistencies with inclusion criteria, sixty-four participants were excluded. Thus, the study ultimately focused on 524 subjects (144 men and 380 women).

### Measures

The questionnaire form consisting of four parts was used to evaluate the relationship between SM use and eating behaviour. The initial section of the questionnaire consisted of demographic characteristics. Subsequently, the second section explored participants’ dietary practices, including meal frequency, meal skipping tendencies, appetite status, dietary preferences and anthropometric measurements. This section also entailed participants’ self-reported body weight and height. The third section delved into participants’ SM usage patterns and their potential tendencies towards addiction. Finally, the fourth section of the questionnaire was dedicated to evaluating participants’ emotional appetite.

### Social media usage and social media addiction

The inquiry concerning SM usage encompassed various dimensions, including the extent and frequency of Internet and SM engagement within a given day, preferred SM tools, and the frequency and duration of interactions with distinct SM tools. Bergen Social Media Addiction Scale (BSMAS) which was developed by Andreassen et al.^(7)^ was used to measure SM addiction. A high score in this questionnaire indicates high SM addiction, while a low score indicates low addiction. Turkish validation of the scale was conducted by Demirci^(8)^. Within the present study’s sample, the BSMAS demonstrated high internal consistency, indicated by a Cronbach’s *α* coefficient of 0·82.

### Emotional appetite assessments

Emotional Appetite Questionnaire (EMAQ) was used to evaluate the emotional appetite of the participants. The EMAQ shows the propensity to eating as a response to positive and negative emotions and situations, developed by Nolan et al.^(9)^. Demirel et al.^(10)^ conducted the validity and reliability of the scale in Turkish. The presence of emotional eating is evaluated with fourteen items in negative/positive emotions and eight items in negative/positive situations. The total positive EMAQ score (EMAQ-P) was obtained by averaging positive emotion and situation scores. Likewise, the total negative emotion and situation scores were averaged to obtain total negative EMAQ score (EMAQ-N). Higher scores were representer of increased emotional eating^(9)^. Cronbach’s *α*s were 0·92 for EMAQ-N and 0·93 for EMAQ-P, indicating high internal consistency in present study.

### Statistical analyses

All statistical analyses were performed using SPSS 25. Data were screened for normality according to Kolmogorov–Smirnov test. All dependent variables were normally distributed. The differences in BMI, BSMAS and EMAQ by SM access frequency were analysed by performing ANOVA, and the significance of the difference between groups was determined by Tukey’s multiple comparison test. To examine the relationship between the duration of SM use, BSMAS, BMI and EMAQ, multiple regression was performed after it was checked whether the statistical assumptions for regression were met. Statistically, the lowest level of significance was accepted as *P* < 0·05.

## Results

### Participant characteristics and Internet, social media usage duration, Bergen Social Media Addiction Scale and Emotional Appetite Questionnaire score

Characteristics of the subjects are presented in Table 1. The mean BMI was 22·2 ± 3·8 kg/m^2^, and most participants (67·4 %) had normal BMI (18·5–24·9 kg/m^2^). While the mean Internet usage duration was 6·6 ± 3·4 h, the mean SM usage duration was 3·2 ± 2·2 h in a day. Moreover, the mean of BSMAS and EMAQ of participants are presented in Table 1.

**Table 1:** Sample descriptives

	Freq	Percent	Mean	sd
Participants	524	100		
Woman	380	72·5		
Men	144	27·5		
Age			21	1·9
Marital status				
Single	519	99		
Married	4	0·8		
Divorced	1	0·2		
Smoker				
Never smoke	377	71·9		
Current smoker	99	18·9		
Used to smoke	48	9·2		
Diet	71	13·5		
Exercise	136	26·0		
Exercise time (h/d)			39·3	32·2
BMI			22·2	3·8
Underweight	69	13·2		
Normal	353	67·4		
Overweight	77	14·7		
Obese	25	4·8		
Total Internet usage duration (h/d)			6·6	3·4
Total SM usage duration (h/d)			3·2	2·2
BSMAS			16·1	5·9
EMAQ-P			4·4	1·9
EMAQ-N			3·1	1·2

SM, social media; BSMAS, Bergen Social Media Addiction Scale; EMAQ, Emotional Appetite Questionnaire; EMAQ-P, positive EMAQ score; EMAQ-N, negative EMAQ score.

### The differences between BMI, social media addiction and emotional appetite according to the frequency of the most accessed social media tools

YouTube (92·6 %), Instagram (90·3 %), Google+ (56·7 %) and Twitter (54·8 %) emerged as the predominant SM tools, each accessed at least once a week, as depicted in online supplementary material, Supplementary Figure 1. Table 2 presents variations in BMI, BSMAS and EMAQ scores based on the frequency of SM access across four commonly used SM platforms. Notably, individuals who primarily used YouTube had the highest averages for BMI, BSMAS and EMAQ-P scores. BSMAS scores increased with higher frequency of Instagram access, but no significant variations were observed in EMAQ-P or EMAQ-N scores. Participants accessing Twitter less than once a week or not at all had significantly lower mean BSMAS scores compared with others. However, no significant differences were found in other scales based on the frequency of accessing Twitter or Google+.

**Table 2:** Differences in BMI, BSMAS and EMAQ by SM accessed frequency

		Less than once a week or none		1–6 times a week		1–9 times a day		10+ times a day		*P*
		Mean	sd	Mean	sd	Mean	sd	Mean	sd	
YouTube Instagram Google + Twitter	BMI	21·3	2·8*	21·7	4·3*	22·1	3·6*	23·4	4·0^†^	**0·003**
	BSMAS	13·9	5·9*	16·2	5·7* ^,^ ^†^	15·8	5·8*	17·8	6·1^†^	**0·002**
	EMAQ-P	3·6	2·1*	4·0	1·9*	4·6	1·8^†^	4·4	2·1* ^,^ ^†^	**0·001**
	EMAQ-N	2·8	1·5	2·8	1·4	3·2	1·6	2·9	1·6	0·063
	BMI	21·0	2·6	23·0	4·9	22·5	3·8	22·0	3·8	**0·036**
	BSMAS	13·3	6·3*	15·5	4·6*	15·3	5·3*	18·2	6·3^†^	**<0·001**
	EMAQ-P	4·2	2·0	4·3	2·0	4·4	1·9	4·5	2·0	0·752
	EMAQ-N	3·2	1·6	2·9	1·5	3·0	1·5	3·2	1·7	0·557
	BMI	22·6	3·9	22·7	4·3	21·8	3·6	21·8	3·2	0·113
	BSMAS	15·6	6·1	15·8	4·9	16·4	6·0	17·5	5·9	0·103
	EMAQ-P	4·5	1·9	4·2	1·7	4·5	2·0	4·3	1·9	0·731
	EMAQ-N	3·2	1·5	2·9	1·5	3·0	1·7	2·9	1·5	0·337
	BMI	22·3	3·9	21·7	4·5	22·2	3·3	22·4	3·9	0·721
	BSMAS	14·7	5·9*	17·1	5·4^†^	16·7	5·5^†^	18·9	6·2	**<0·001**
	EMAQ-P	4·3	1·9	4·6	1·8	4·4	2·0	4·5	1·9	0·807
	EMAQ-N	3·1	1·5	2·9	1·3	3·1	1·6	3·2	1·8	0·767

BSMAS, Bergen Social Media Addiction Scale; EMAQ, Emotional Appetite Questionnaire; SM, social media; EMAQ-P, positive EMAQ score; EMAQ-N, negative EMAQ score.

*P* values were calculated by ANOVA. Bold indicates statistically significant difference (*P* < 0.05).

* Different lower case (*-†) in the same row means the statistically significant difference among the values, according to the *post hoc* Tukey’s test (*P* < 0·05), the same lower case (*-†) means that there was not statistically difference (*P* > 0·05).

† Different lower case (*-†) in the same row means the statistically significant difference among the values, according to the *post hoc* Tukey’s test (*P* < 0·05), the same lower case (*-†) means that there was not statistically difference (*P* > 0·05).

A multiple linear regression analysis was conducted to assess whether certain independent variables could predict the EMAQ scores (Table 3). For EMAQ-P, the overall model was statistically significant, indicating that the predictor variables explained about 1 % of the variance in EMAQ-P scores. Specifically, the BSMAS variable significantly contributed to the model, while SM usage duration and BMI did not. Similarly, for EMAQ-N, the regression equation was significant, with the predictor variables explaining about 5 % of the variance. In this case, both BSMAS and BMI were found to contribute to the model, whereas SM usage duration did not.

**Table 3:** Multiple regression analysis results for predicting EMAQ-P and EMAQ-N

Variables	Categories	*B*	s e	*β*	*t*	*P*	95 % CI		Adj. R^2^	*F*	*P*
							Lower	Upper			
EMAQ-P	Constant	4·037	0·575		7·025	<0·001	2·908	5·167	0·010	2·652	**0·048**
	SM usage time	−0·036	0·042	−0·042	−0·087	0·384	−0·118	0·046			
	BSMAS	0·043	0·016	0·132	2·763	**0·006**	0·012	0·074			
	BMI	−0·009	0·023	−0·017	−0·386	0·700	−0·053	0·036			
EMAQ-N	Constant	1·297	0·455		2·851	0·005	0·403	2·191	0·046	9·205	**<0·001**
	SM usage time	−0·036	0·033	−0·050	−1·080	0·281	−0·100	0·029			
	BSMAS	0·058	0·012	0·221	4·728	**<0·001**	0·034	0·083			
	BMI	0·043	0·018	0·105	2·427	**0·016**	0·008	0·078			

EMAQ, Emotional Appetite Questionnaire; EMAQ-P, positive EMAQ score; EMAQ-N, negative EMAQ score; *B*, unstandardised regression coefficient; se, unstandardised standard error; *β*, standardised regression coefficient; *t*, *t*-test value; SM, social media; BSMAS, Bergen Social Media Addiction Scale. Bold indicates statistically significant difference (*P* < 0.05).

## Discussion

The global daily engagement with SM platforms is continually expanding. This aligns with our findings, which parallel those of Statista^(11)^ indicating that Internet users devoted an average of 2 hours and 24 min per d to SM in 2020. The escalating online activity is often associated with problematic SM usage, potentially indicative of incipient addiction. Such an inclination can contribute to challenges in emotion regulation^(12,13)^.

Various studies have posited a potential link between SM addiction and disruptions in emotional regulation^(14,15)^. In the present study, emotional appetite was assessed using the EMAQ, revealing a mean EMAQ-P score of 4·4 ± 1·9 and an EMAQ-N score of 3·1 ± 1·2. These findings corroborate earlier reports^(12,16)^.

The relationship between SM addiction and emotional appetite remains relatively underexplored within the existing literature. Solely, one study reported the association between addictive phone use and dysregulated eating and food addiction^(17)^. Positive relations between SM addiction and emotional dysregulation were observed previously^(18,19)^. Given the common phenomenon of altered eating behaviours serving as coping mechanisms among young adults, it is plausible that both SM addiction and heightened emotional appetite could be precipitated by emotional dysregulation and evolving coping strategies within this demographic.

Statista^(20)^ reported that Facebook was the most popular social network worldwide in 2020, while YouTube was the second. Interestingly, YouTube and Instagram were found as the most frequently used SM tools among the participants of the current study. However, Nelson and Fleming^(21)^ reported that the most frequently used SM tools of young participants in the USA were Facebook, Snapchat, Instagram, and YouTube, while according to Jairoun and Shahwan’s study^(22)^ Instagram and Snapchat were the most popular in the UAE.

Researchers point out that the associations between SM and worse well-being were associated with the exposure to visual and image-oriented SM rather than the general use of SM tools^(23,24)^. Likewise, within this study, participants who primarily accessed YouTube demonstrated elevated scores in BMI, BSMAS and EMAQ-P. A plausible rationale for these findings could be attributed to the potential impact of YouTube video content on eating behaviours. For example, recent research highlighted a positive link between watching mukbang videos on SM and disordered eating as well as Internet addiction among young adults^(25)^. In the current study, a positive correlation was observed between SM addiction and the frequency of participants’ Instagram use, while no significant variance was found in emotional appetite scores based on Instagram access. Notably, these findings diverge from certain research that has suggested a connection between Instagram and eating disorders^(26,27)^, although a recent systematic review noted an inconclusive relationship^(28)^.

The study found no significant differences in BMI, SM addiction or emotional appetite based on the frequency of accessing Twitter or Google+. Notably, participants accessing Twitter less than once a week or not at all had significantly lower BSMAS scores. While there is no direct literature linking Twitter to eating behaviour or disorders, some studies suggest a correlation between posting food-related content on Twitter and problematic eating behaviours^(29,30)^.

BSMAS was found as a predictor for EMAQ-P, whereas SM usage and BMI were found not to be significant predictors. Considering EMAQ-N, it was found that BSMAS and BMI were significant predictors while SM usage duration was not a predictor for EMAQ-N. This study provides preliminary evidence demonstrating the emotional appetite risks associated with SM addiction. Problematic SM usage and being addicted to SM might have complicated effects on emotional appetite.

## Strengths and limitations

To our knowledge, this research provides the first report on the association between SM addiction and emotional appetite. This study has several strengths. To start, the BSMAS has not been previously used in studies investigating SM use and emotional appetite. Additionally, the incorporation of validated and reliable scales to gauge emotional appetite enhances the study’s methodological robustness. However, the online modality employed for data collection presents a limitation, as it contrasts with traditional face-to-face interactions. Furthermore, a gender imbalance in the participant pool affects the study’s generalisability. Additionally, the absence of dietary intake and food choice data precludes the evaluation of participants’ energy and nutrient intake as well as their dietary preferences.

### Conclusion

The results of the present study provide initial evidence for the interaction between negative and positive emotional eating and SM engagement and addiction. Notably, having SM addiction and extended SM usage duration had the effect on both negative and positive emotional appetite. Considering the widespread use of SM and its potential impact, further research is required for comprehensive investigations to examine the influences of SM addiction on eating behaviours.

## Supporting information

Sevim et al. supplementary material Sevim et al. supplementary material

## Supplementary material

For supplementary material accompanying this paper visit https://doi.org/10.1017/S1368980024000466.
