# Supplementary material for: The relationship between social media addiction and emotional appetite: a cross-sectional study among young adults in Turkey
Source: Public Health Nutr. 2024 Feb 15;27(1):e72. doi: 10.1017/S1368980024000466 (PMC10966846; doi:10.1017/S1368980024000466)
Supplement: Sevim et al. supplementary material [file S1368980024000466sup001.docx]

Supplementary Figure 1. The frequency of access to social media tools (percentage)
